# Comparative Study of Levobupivacaine Versus Levobupivacaine With Dexmedetomidine as an Adjuvant in Transversus Abdominis Block for Postoperative Pain Relief in Abdominal Hysterectomy Patients: A Randomized, Double-Blind Study

**DOI:** 10.7759/cureus.59523

**Published:** 2024-05-02

**Authors:** Romi Romi, Wasimul Hoda, Sourabh Kumar, Bharati Bharati, Saurabh Toppo, Dipali Singh, Priyanka Oraon, Reena Kumari, Alka Lakra, Shio Priye

**Affiliations:** 1 Anaesthesiology, Rajendra Institute of Medical Sciences, Ranchi, IND; 2 Anaesthesiology, Bhagwan Mahavir Medica Superspecialty Hospital, Ranchi, IND; 3 Superspeciality Anaesthesiology, Rajendra Institute of Medical Sciences, Ranchi, IND

**Keywords:** postoperative pain, abdominal hysterectomy, transversus abdominis block, adjuvant, dexmedetomidine, levobupivacaine

## Abstract

Background

Adjuvants have been discovered to prolong the analgesic impact of local anesthetics (LA), while the transversus abdominis plane (TAP) block offers sufficient postoperative pain relief after various abdominal procedures. Nevertheless, the impact of the given LA will determine the duration of the TAP block. Thus, in this investigation, we aimed to estimate the analgesic impact of combining dexmedetomidine (DEX) with levobupivacaine in the TAP block for patients having an infraumbilical incision for an abdominal hysterectomy while under spinal anesthetic.

Aim

This study aimed to determine the analgesic effect of DEX (0.5 mcg/kg) to 20 ml of 0.25% levobupivacaine on each side in the TAP block in patients undergoing total abdominal hysterectomy (TAH).

Materials and methods

Two groups of 30 patients each, with ASA grades 1 and 2, were randomly selected from patients. Group N, comprising 30 patients, had a bilateral TAP block using 2 mL of normal saline and 20 mL of 0.25% levobupivacaine. Group D (n = 30 patients) was given DEX at a dose of 0.5 mcg/kg (2 mL) in addition to 20 mL of 0.25% levobupivacaine given bilaterally. The TAP block was administered just after skin closure. Time of the initial analgesic dose administration; total fentanyl doses utilized as rescue analgesia; pain scores (numerical rating scale (NRS)) at 2, 4, 6, 8, 12, and 24 hours; and pre- and postoperative cortisol levels were also noted. For each group, 1 gram IV paracetamol was administered every eight hours. Drugs used for rescue analgesia (RA) were diclofenac 75 mg IV stat and fentanyl 1 mcg/kg.

Results and discussion

In addition to Group N having lower NRS scores at rest, Group D had a considerably longer time for initial rescue analgesia than Group N. There was also a significant decrease in the total fentanyl consumption and postoperative serum cortisol levels in Group D in contrast to Group N.

Conclusion

Potential adjuvant DEX prolongs postoperative analgesia in patients experiencing abdominal hysterectomy when used alongside LA in TAP.

## Introduction

The paramount importance of effective pain management for surgical patients cannot be overstated. Proper pain relief yields significant physiological benefits, whereas inadequate postoperative pain control can result in a range of undesirable consequences, including discomfort experienced by the patient, prolonged immobility, thromboembolic events, and pulmonary problems. Multimodal analgesia is advised for postoperative pain control in contemporary surgical practice in an effort to reduce dependency on opioids, which have negative side effects. One such component of multimodal analgesia for abdominal surgeries is the transversus abdominis plane (TAP) block. This method effectively relieves postoperative pain by targeting the innervation of the anterolateral abdominal wall, which extends from T6 to L1 [1,2]. However, the effects of the local anesthetics (LA) that are provided limit how long the pain relief lasts. To extend the duration of TAP block effectiveness, infusion catheters can be employed. TAP blocks find utility in a diversity of surgical settings, such as open, laparoscopic abdominal procedures and inpatient and outpatient surgeries. These blocks are generally administered intraoperatively, either prior to the surgical incision or following the procedure, just as the patient emerges from anesthesia. The TAP block's effectiveness lies in the even dispersion of LA within the interfacial plane. Recent innovations involve the addition of adjuvants, such as dexmedetomidine (DEX), to LA, aiming to extend the impact of TAP blocks [3-8]. DEX, a selective α2 (alpha 2) adrenergic agonist [9] with analgesic and sedative characteristics, has demonstrated increased efficacy when used in conjunction with levobupivacaine [10] via epidural or intrathecal routes. However, limited research has explored its potential benefits in combination with TAP blocks.

The two primary components of the stress response following surgery are inflammatory-immune and neuroendocrine-metabolic. The extent of the body's integrated reaction to stress is largely dependent on the type, extent, and length of surgery. Perioperative stress response is modulated by various anesthetic agents: IV and volatile anesthetic agents - propofol (1-2 mg kg^−1^) as an induction dosage reduces circulating cortisol levels but does not stop aldosterone and cortisol release. Etomidate reduces adrenocortical activity by reversibly suppressing the enzymes 11β-hydroxylase and 17α-hydroxylase. After a single induction dose of 0.3 mg kg^−1^, etomidate suppresses the production of cortisol and aldosterone for up to eight hours. Compared to IV anesthetics like propofol plus remifentanil, volatile anesthetics suppress catecholamines, growth hormone (GH), cortisol, and adrenocorticotropic hormone (ACTH) more strongly [11]. Sevoflurane significantly decreases ACTH, cortisol, and GH concentrations during laparoscopic surgery in contrast to isoflurane [12].

Analgesics and other medications - benzodiazepines, such as “midazolam (0.2 to 0.4 mg kg^−1^) or infusion (0.9 to 0.125 mg kg^−1^h^−1^), prevent the hypothalamic-pituitary content of the HPA axis from producing cortisol [13]. Both limb and abdominal surgeries have been shown to have this impact.

Clonidine and DEX, two centrally acting α2-adrenoceptor agonists, inhibit the sympathetic nervous system (SNS)-mediated surgical stress response. The central sympathetic output is decreased when α2-receptors in the lateral reticular nucleus are stimulated. Stimulation of α2-receptors increases endogenous opioid secretion and changes the descending pathways in the spinal cord that are included in spinal nociceptive processing. These processes help to lessen the cardiovascular and sympathoadrenal reactions to surgical stimulation. DEX reduces cortisol and renin concentrations, which imparts hemodynamic stability and impaired pancreatic insulin secretion [13].

Local anesthetics combined with wide-ranging neuraxial analgesia prevent the body's hormones and metabolism from reacting negatively to the lower limb and pelvic surgery. By preventing the afferent stimulation of the hypothalamus and the efferent stimulation of the liver, and adrenal glands, as well as the pancreas, neuraxial epidural and spinal anesthesia inhibit the HPA axis response. It also leads to impaired production of cortisol, adrenaline, GH, and adrenocorticotropic hormone [11-13].

This prospective, double-blinded, randomized research seeks to evaluate the analgesic impact and changes in postoperative serum cortisol levels after incorporating DEX into levobupivacaine for TAP blocks in patients going through abdominal hysterectomy. Our primary objective is to determine the analgesic effect of adding DEX (0.5 mcg/kg) to 20 mL of 0.25% levobupivacaine on each side of the TAP block in patients undergoing TAH. Secondary objectives are as follows: a) to investigate the changes in cortisol levels in the patients preoperatively (one day before surgery) and postoperatively (after 12 hours of surgical incision) after giving the TAP block; b) to identify and monitor any potential complications or side effects.

## Materials and methods

This is a prospective double-blinded randomized control study. The duration of the study is one year from the time of approval by the research and ethics committee. The Institutional Ethical Council gave its clearance before the prospective study could be carried out in the Anesthesiology Department of the Rajendra Institute of Medical Sciences in Ranchi, India. Sixty American Society of Anesthesiologists (ASA) grade I and II patients in the age category of 18-65 years and weight between 50 and 65 kgs in both study groups were taken for abdominal hysterectomies, and their written informed consent was obtained (CTRI registration no. CTRI/2022/04/041932).

Method of randomization

Using the "closed envelope method," the patients were split into two categories of 30 each at random: 1) Group N (n = 30 patients) had a TAP block with 38 mL of 0.25% levobupivacaine and 2 mL of normal saline. The total volume (40 mL) was divided equally (20 mL) and administered bilaterally. 2) Group D (n = 30 patients) got 38 mL of 0.25% levobupivacaine along with 2 mL of 0.5 mcg/kg inj. DEX. The total volume (40 mL) was divided equally (20 mL) and administered bilaterally.

Recorded were the first analgesic administration time, the total number of rescue analgesic doses utilized, pain scores (NRS), pre-and postoperative cortisol levels, and side effects. One gram paracetamol IV eight hourly was given to both groups.

Drugs used for rescue analgesia (RA) were diclofenac 75 mg IV stat and then fentanyl 1 mcg/kg.

Inclusion criteria

The inclusion criteria were as follows: patient’s consent, the patient posted for a total abdominal hysterectomy, age group 18-65 years, weight 50-65 kgs, and ASA physical statuses I and II.

Exclusion criteria

The exclusion criteria were as follows: refusal of the patient; presence of comorbidities, like severe anemia, uncontrolled diabetes mellitus, asthma, hypertension, cardiac disease, and coagulopathies; patients classified as III and above in the ASA; drug allergy; and local site infection.

Procedure

Patient selection was randomized using a closed-envelope method to ensure a random distribution. This study included patients undergoing abdominal hysterectomy under spinal anesthesia (employing 3 mL of 0.5% heavy bupivacaine). After obtaining informed written consent and properly identifying the patients, a TAP block was done on each side following skin closure.

The landmark-guided TAP block entrance is located at Petit's lumbar triangle, which is positioned between the iliac crest and lower costal margin. The latissimus dorsi muscle borders this anatomical area from its posterior side, the external oblique muscle from the anterior, and the iliac crest inferiorly (the base of the triangle). A 22 G blunt needle (modified by blunting the tip with scissors) was inserted into the triangle of Petit. The internal and external oblique muscles popped twice when the needle went through them. The usage of the blunt needle during LA injection was chosen to enhance the perception of loss of resistance. After injecting the drug and confirming proper needle placement in the TAP through a backflow check following syringe disconnection, 20 ml of 0.25% levobupivacaine and 2 ml of normal saline were bilaterally administered in the patients of Group N.

Group D, which consisted of 30 patients, on the other hand, received 20 ml of 0.25% levobupivacaine bilaterally along with 0.5 mcg/kg (2 mL) of DEX. Patient vital signs, namely, heart rate, ECG, non-invasive blood pressure, and oxygenation (SpO_2_) as recommended by ASA monitoring standards, were continuously monitored throughout the procedure to detect any hemodynamic changes resulting from the administration of levobupivacaine and DEX.

Observations

Numerical rating scale (NRS) assessments were conducted on a scale from 0 to 10, where 0 denoted no pain and 10 indicated an absolute worst kind of pain. NRS scores were recorded at two, four, six, eight, 12, and 24 hours after the administration of the TAP block in both groups. In both study groups, the time of request for the first RA was recorded. The interval between the administration of the first and second rescue analgesics was recorded in each group. The total fentanyl consumption was recorded in both study groups.

Comparison of serum cortisol levels preoperatively (one day before surgery) and postoperatively (12 hours after skin incision) to assess stress levels in the patients and comparison of any potential adverse effects were performed.

Statistical analysis

IBM SPSS Statistics for Windows, version 28.0 (released 2021, IBM Corp., Armonk, NY) was utilized to conduct statistical analysis. Outcomes were shown as mean and standard deviation (SD) for continuous variables and as a proportion for categorical variables. The significance of two means evaluated by Chi-square and unpaired “t” test for categorical data. A P < 0.05 is regarded as statistically significant. The sample size for each group is estimated to be 27.23-30 by employing the formula: -n = [P1(1 - P1) + P2 (1 - P2)]/ (P1-P2)2 × F. 

Here, n denotes the sample size per group, P1 = 35% or 0.35, P2= 75% or 0.75”(23), and F= 7.9 for 80% power and 10.5 for 90%.

## Results

**Figure 1:**
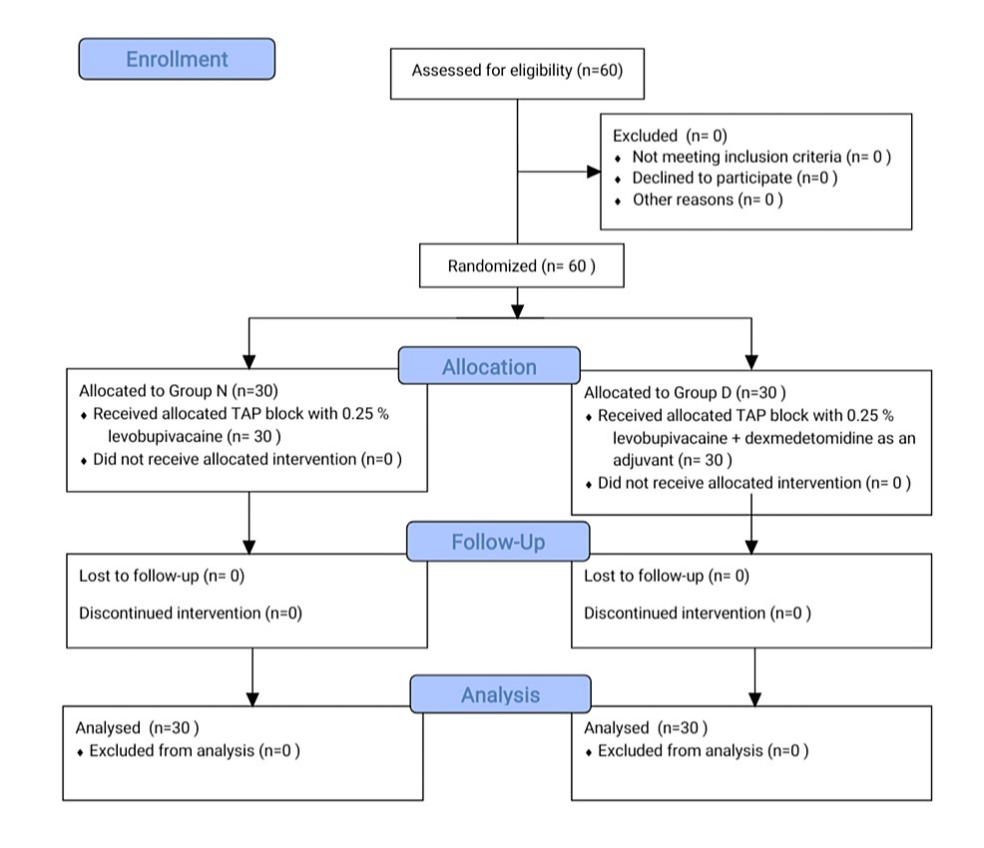
CONSORT flow diagram

Sixty individuals were enrolled in the study, and they underwent several types of evaluation. As shown in Figure 1, our study perfectly complies with the CONSORT recommendations.

Table 1 shows the demographic details of patients in both study groups. In Group N, the mean age of the patients was 42.03 years with a standard deviation of ±4.005. Conversely, in Group D, the mean age was 41.06 years with a standard deviation of ±6.001. In Group N, the mean weight of patients was 55.23 kgs with a standard deviation of ±4.001. Conversely, in Group D, the mean age was 53.04 kgs with a standard deviation of ±4.005. Regarding age, weight, and ASA grade, there were no statistically significant variations between the demographic characteristics of the two study groups.

**Table 1:** Enrolled patients' demographic details (n = 30 in each group) ASA: American Society of Anaesthesiologists. The data are presented as mean ± SD (standard deviation) or n (%).

Parameters	Group N	Group D
Age (years)	42.03 ± 4.005	41.06 ± 6.001
Weight (kg)	55.23 ± 4.001	53 .04± 4.005
ASA classification		
I	23 (76.66 %)	22 (73.33 %)
II	7 (23.33 %)	8 (26.66 %)

**Figure 2:**
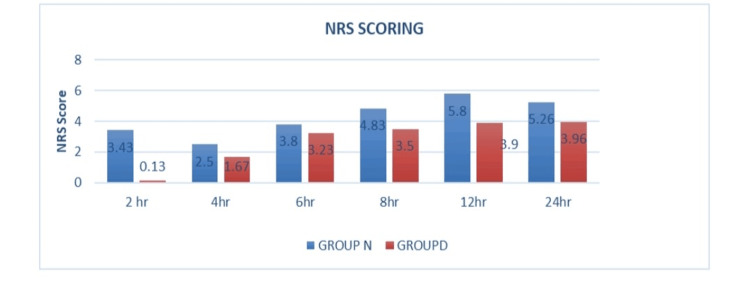
Numerical rating scale (NRS) pain scores in both scores

The NRS scores were monitored in both study groups over a period of 24 hours, assessing pain levels experienced by the patients postoperatively. The NRS pain scores in both groups are visually represented in Figure 2. As compared to Group N, Group D's NRS scores were noticeably lower.

Table 2 shows the comparison of NRS pain scores in both groups. The NRS pain scores are lower in Group D than in Group N, which are statistically significant at two, four, eight, 12, and 24 hours but non-significant at six hours postoperatively.

**Table 2:** Comparison of numerical rating scale (NRS) pain scores in both groups

Time (postoperative)	NRS pain scores		p-value
	Group N	Group D	
2 hours	3.4 ± 1.3	0.13 ± 0.3	<0.0001
4 hours	2.5 ± 1.3	1.6 ± 0.9	0.0028
6 hours	3.8 ± 2	3.2 ± 1.1	0.15
8 hours	4.8 ± 1.4	3.5 ± 1.5	0.001
12 hours	5.8 ± 1.2	3.9 ± 1.4	<0.0001
24 hours	5.2 ± 1.6	3.9 ± 0.9	0.0003

**Figure 3:**
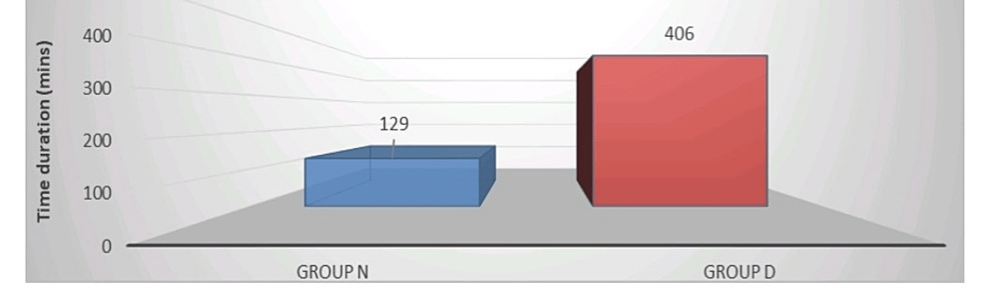
Time of request for the first rescue analgesia in both groups

Figure 3 depicts the visual representation of the time of request for the first RA in both study groups. The drug used for the first RA is Inj. diclofenac 75 mg IV stat. The time of request for the first RA was longer in Group D (406 ± 87.3 minutes) in comparison to Group N (129 ± 27.8 minutes).

**Figure 4:**
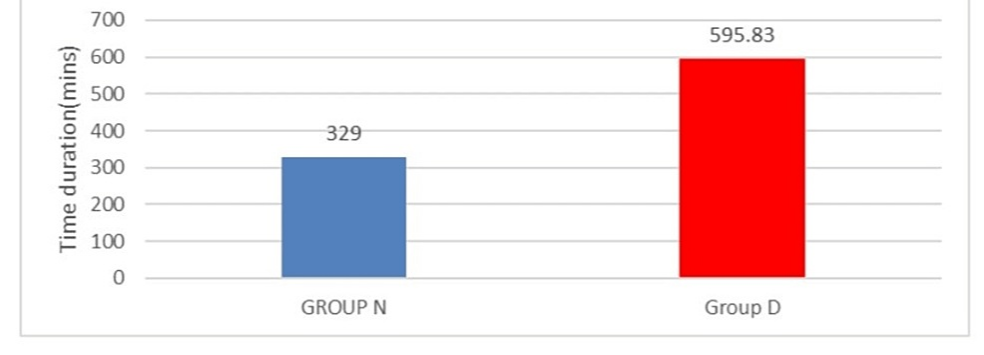
Time of request for the second rescue analgesia in both groups

Figure 4 depicts the visual representation of the time of request for the second RA in both study groups. The drug used for the second RA is Inj. fentanyl 1 mcg/kg IV in both study groups. The time of request for the second RA was longer in Group D (595.8 ± 14.1 minutes) in comparison to Group N (329 ± 49.57 minutes).

**Figure 5:**
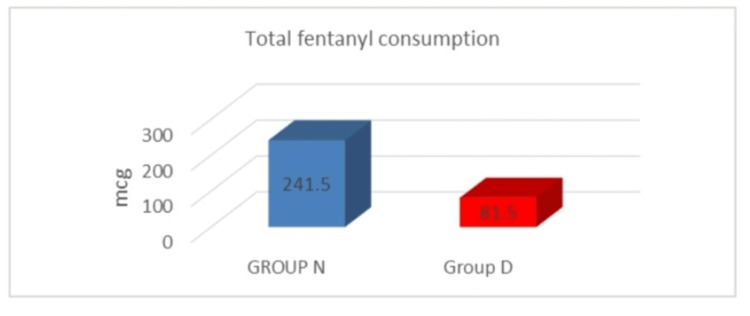
Total fentanyl consumption in both groups

The visual representation of the total fentanyl consumption in both study groups is shown in Figure 5. Group D had a considerably lower total fentanyl intake (81.5 ± 29.27 mcg) than Group N (241.5 ± 59.67 mcg) in 24 hours postoperatively.

Table 3 depicts the comparison of analgesic requirements in both study groups. The time of request for the first RA was significantly (p < 0.001) longer in Group D (406 ± 87.31 minutes) in comparison to Group N (129 ± 27.86 minutes). The time of request for the second RA was also longer in Group D (595.8 ± 14.1 minutes) in comparison to Group N (329 ± 49.57 minutes), which was statistically significant (p < 0.001). The total fentanyl intake in Group D was much lower (81.5 ± 29.27 mcg) than in Group N (241.5 ± 59.67 mcg), and the result was statistically significant (p < 0.001). In our study, the addition of DEX to levobupivacaine as an adjuvant in the TAP block prolongs postoperative analgesia duration in patients undergoing abdominal hysterectomy as compared to when levobupivacaine is used alone. 

**Table 3:** Comparison of analgesic requirements in both groups

Analgesic requirements	Group N	Group D	P value
Time for the first rescue analgesia (minutes)	129 ± 27.86	406 ± 87.31	<0.001
Time for the second rescue analgesia (minutes)	329 ± 49.57	595.8 ± 114.1	<0.001
Total fentanyl consumption (mcg)	241.5 ± 59.67	81.5 ± 29.27	<0.001

**Figure 6:**
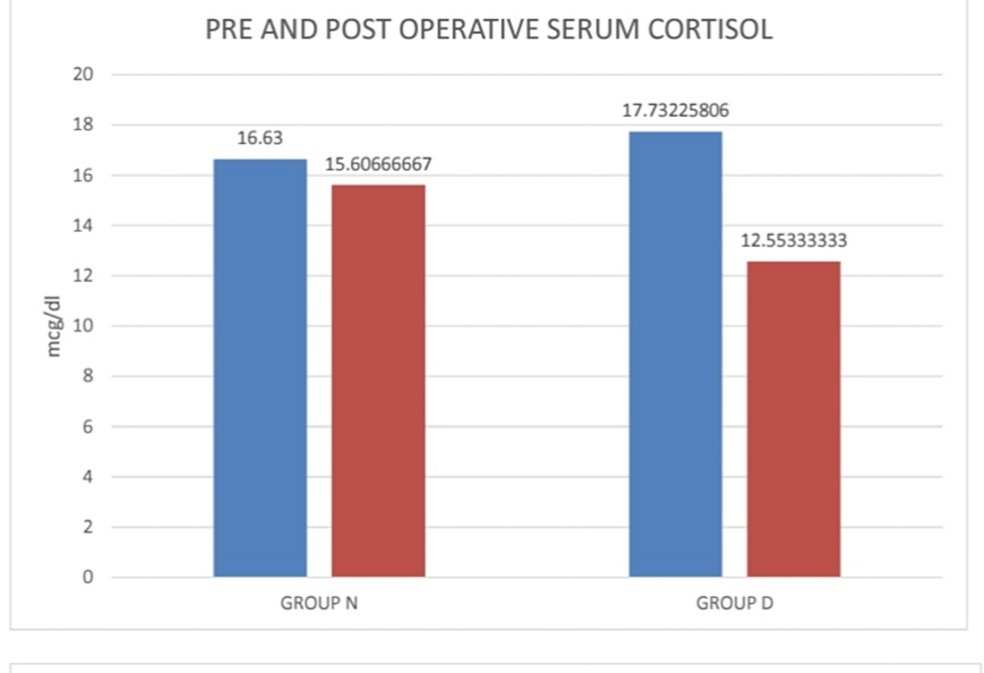
Pre- and postoperative cortisol levels in both groups Preoperative: one day before surgery, postoperative: 12 hours after skin incision

Figure 6 depicts the visual representation of pre- and postoperative serum cortisol levels to assess the stress levels of patients in both study groups. In Group N, the preoperative serum cortisol level was 16.63 ± 3.54 mcg/dl and the postoperative serum cortisol level was 15.6 ± 3.65 mcg/dl. In Group D, the preoperative serum cortisol level was 17.73 ± 2.92 mcg/dl, and the postoperative serum cortisol level was 12.55 ± 2.65 mcg/dl. There was a reduction in the postoperative serum cortisol levels in both groups. 

Table 4 shows the comparison of the pre- and post-operative serum cortisol levels of patients in both study groups. It shows a reduction in the postoperative serum cortisol levels in both groups. However, it was not significant in Group N, while in Group D, the reduction was significant (P value < 0.001). The addition of DEX definitely reduced stress levels in Group D by increasing the analgesic duration and by decreasing the fentanyl demand as RA. 

**Table 4:** Comparison of serum cortisol levels in both groups Preoperative: one day before surgery, postoperative: 12 hours after skin incision

Serum cortisol (mcg/dl)	Group N	Group D
Preoperative	16.63 ± 3.54	17.73 ± 2.92
Postoperative	15.6 ± 3.65	12.55 ± 2.65
p-value	0.2718	<0.0001

Table 5 shows the comparison of adverse effects in both study groups. Adverse effects, like hypotension, bradycardia, shivering, pruritus, and postoperative nausea vomiting (PONV), were included and compared. The incidence was shown in the number of patients and their percentage in the study group. There were no significant hemodynamic changes or adverse impacts in either group.

**Table 5:** Comparison of adverse effects in both groups PONV: postoperative nausea vomiting. Information is presented as n (%).

Adverse events	Group N		Group D	
	No.	%	No.	%
Hypotension	3	10	2	6.6
Bradycardia	1	3.3	1	3.3
Shivering	4	13.3	4	13.3
Pruritus	0	0	0	0
PONV	1	3.3	0	0

## Discussion

This study's findings underscore the significant benefits of incorporating DEX as an adjunct to levobupivacaine in landmark-guided bilateral transversus abdominis (TrA) blocks for patients undergoing TAH surgery with infra-umbilical incisions. This strategy led to a significant drop in postoperative cortisol levels and a reduction in the overall amount of fentanyl consumed and postoperative pain levels. The interval until the first RA requirement was extended, and the NRS scores were lower when DEX was introduced alongside levobupivacaine. It is noteworthy that patients receiving the TAP block with levobupivacaine and DEX experienced a significantly prolonged duration of analgesia, in contrast to those receiving the TAP block with levobupivacaine alone, as the time of request for the first RA was approximately 129 ± 27.86 minutes in Group N, and in Group D, it was approximately 406 ± 87.31 minutes. The time interval between the request for the first and second RA was also much prolonged in Group D than in Group N. Furthermore, the demand for total fentanyl consumption was diminished when compared to cases where only levobupivacaine was utilized.

Salem et al. [14], in their study, reported similar findings, indicating “a significant reduction in total morphine consumption, VAS (visual analog scale) scores, vomiting, and postoperative nausea as well as postoperative cortisol levels when bupivacaine was used with DEX as an adjuvant in bilateral ultrasound-guided rectus sheath blocks. For cancer patients having prolonged midline abdominal incisions, this combination functioned well for managing discomfort following surgery.

In a different trial, Abdelaal et al. [15] examined the efficacy of combining levobupivacaine and DEX in pre-emptive TAP blocks for postoperative pain control following abdominoplasty. The research study comprised 69 patients in total. The findings demonstrated that the groups receiving DEX (Group M) and levobupivacaine (Group L) both had significantly lower pain scores than the control group. In addition, the total 24-hour meperidine consumption in Groups M and L was notably less than the control group, with a statistical significance of P < 0.001. Furthermore, Group M had a lower total meperidine consumption than Group L (P < 0.01).

McDonnell et al. [16] performed a randomized controlled experiment to determine the efficacy of ropivacaine (1.5 mg/kg) TAP blocks in delivering postoperative analgesia within the first 48 hours after the procedure. Fifty women obtaining spinal anesthesia for elective cesarean delivery participated in the study. Patients undergoing AP blocks with ropivacaine showed lower VAS scores, lower total morphine doses, and a lower occurrence of sedation in the first 48 hours postoperatively upon comparison with the placebo group.

Abdallah et al. [17] investigated whether TAP blocks could lower the amount of intravenous morphine used in the first 24 hours after cesarean delivery with a comprehensive review and meta-analysis. Reduction in intravenous morphine intake within the first 24 hours was the main aim; pain scores and adverse effects associated with opioids in both mothers and newborns were the secondary outcomes. Their examination, including a total of 312 patients, demonstrated a decrease in the mean 24-hour morphine consumption in the group receiving TAP blocks without spinal morphine. In addition, TAP blocks were associated with lower VAS pain scores and a reduced frequency of adverse reactions. However, when spinal morphine was included as part of the analgesic plan, no significant differences in the primary and secondary results were observed. These findings suggest that superior-quality analgesia can be achieved with TAP blocks in contrast to placebo, resulting in reduced morphine consumption within the first 24 hours when integrated into a multimodal analgesic regimen that does not include spinal morphine administration.

In our study, the stress levels as assessed by pre- and postoperative cortisol levels were recorded, and it was seen that the postoperative cortisol levels were reduced in both groups. However, in Group D, the reduction in postoperative cortisol levels was statistically significant. It was due to the prolonged analgesic effect in Group D due to DEX use as an adjuvant in TAP block. Moreover, the adverse effects were not significant in both study groups as evidenced in our study.

Marana et al. [12] compared intraoperative and postoperative neuroendocrine stress responses during total intravenous anesthesia (TIVA) using propofol and remifentanil versus sevoflurane anesthesia during laparoscopic surgery and found that TIVA inhibited the ACTH-cortisol axis and reduced NE, E, and GH levels, but it enhanced PRL and had a weak effect on thyroid hormone concentrations as compared to sevoflurane anesthesia, thus supporting our study.

Qin et al. [18] studied the impact of DEX added to ropivacaine for the TAP block on stress response in laparoscopic surgery in a randomized controlled trial and found that the addition of DEX as an adjunct at the dose of 0.5 μg/kg into ropivacaine for ultrasound-guided TAP block is the optimal dose to inhibit stress response with limited impact on blood pressure and heart rate in patients undergoing laparoscopy gynecological surgery.

Furthermore, there are some limitations in our study. First, we only investigated female subjects. Second, the type of surgery was the same in all patients. Third, our study included the use of a landmark-guided technique rather than USG-guided. The USG-guided technique is more efficient. Hence, future studies are needed to evaluate the efficacy of the addition of DEX as an adjuvant to the TAP block for more painful procedures in other types of surgery, in both gender patients and also with the USG-guided technique.

## Conclusions

In our study, when DEX was added to levobupivacaine as an adjuvant in the TAP block, the patients undergoing TAH via infra-umbilical incision experienced longer postoperative analgesia, reduced postoperative opioid consumption, and postoperative serum cortisol levels, which imparts hemodynamic stability than when levobupivacaine was administered alone. Therefore, we found that DEX is a safe and potent adjuvant in the TAP block that might be employed to give better postoperative analgesia and reduce the stress response to surgery and pain postoperatively.
